# High Mobility Group Box 1 and Interleukin 6 at Intensive Care Unit Admission as Biomarkers in Critically Ill COVID-19 Patients

**DOI:** 10.4269/ajtmh.21-0165

**Published:** 2021-05-03

**Authors:** Chaisith Sivakorn, Jutamas Dechsanga, Lawan Jamjumrus, Kobporn Boonnak, Marcus J. Schultz, Arjen M. Dondorp, Weerapong Phumratanaprapin, Ranistha Ratanarat, Thummaporn Naorungroj, Patchrapa Wattanawinitchai, Tanaya Siripoon, Chatnapa Duangdee, Tachpon Techarang

**Affiliations:** 1 Department of Clinical Tropical Medicine, Faculty of Tropical Medicine, Mahidol University, Bangkok, Thailand;; 2 Division of Pulmonary and Critical Care, Department of Medicine, Chonburi Hospital, Chonburi, Thailand;; 3 Division of Pulmonary and Critical Care, Department of Medicine, Buddhasothorn Hospital, Chachoengsao, Thailand;; 4 Department of Microbiology and Immunology, Faculty of Tropical Medicine, Mahidol University, Bangkok, Thailand;; 5 Mahidol–Oxford Tropical Medicine Research Unit, Faculty of Tropical Medicine, Mahidol University, Bangkok, Thailand;; 6 Department of Intensive Care & Laboratory of Experimental Intensive Care and Anesthesiology (L·E·I·C·A), Academic Medical Center, University of Amsterdam, Amsterdam, The Netherlands;; 7 Centre for Tropical Medicine and Global Health, Nuffield Department of Medicine, Oxford University, Oxford, United Kingdom;; 8 Siriraj Hospital, Division of Critical Care, Department of Medicine, Faculty of Medicine, Mahidol University, Bangkok, Thailand;; 9 Hospital for Tropical Disease, Faculty of Tropical Medicine, Mahidol University, Bangkok, Thailand;; 10 School of Medicine, Walailak University, Nakhon Si Thammarat, Thailand

## Abstract

Exuberant inflammation manifesting as a “cytokine storm” has been suggested as a central feature in the pathogenesis of severe coronavirus disease 2019 (COVID-19). This study investigated two prognostic biomarkers, the high mobility group box 1 (HMGB1) and interleukin-6 (IL-6), in patients with severe COVID-19 at the time of admission in the intensive care unit (ICU). Of 60 ICU patients with COVID-19 enrolled and analyzed in this prospective cohort study, 48 patients (80%) were alive at ICU discharge. HMGB1 and IL-6 plasma levels at ICU admission were elevated compared with a healthy control, both in ICU nonsurvivors and ICU survivors. HMGB1 and IL-6 plasma levels were higher in patients with a higher Sequential Organ Failure Assessment (SOFA) score (> 10), and the presence of septic shock or acute kidney injury. HMGB1 and IL-6 plasma levels were also higher in patients with a poor oxygenation status (PaO_2_/FiO_2_ < 150 mm Hg) and a longer duration of ventilation (> 7 days). Plasma HMGB1 and IL-6 levels at ICU admission also correlated with other prognostic markers, including the maximum neutrophil/lymphocyte ratio, D-dimer levels, and C-reactive protein levels. Plasma HMGB1 and IL-6 levels at ICU admission predicted ICU mortality with comparable accuracy to the SOFA score and the COVID-GRAM risk score. Higher HMGB1 and IL-6 were not independently associated with ICU mortality after adjustment for age, gender, and comorbidities in multivariate analysis models. In conclusion, plasma HMGB1 and IL6 at ICU admission may serve as prognostic biomarkers in critically ill COVID-19 patients.

## INTRODUCTION

Coronavirus disease 2019 (COVID-19) has emerged as a major threat worldwide, affecting more than 140 million people and resulting in more than 3 million deaths worldwide as of early April 2021.^1^ The clinical course of COVID-19 varies substantially among patients. Most infected individuals remain asymptomatic or exhibit only mild to moderate symptoms; approximately 15% progress to severe pneumonia, and up to 5% may eventually need admission to the intensive care unit (ICU) due to acute respiratory distress syndrome (ARDS), shock, or multiple organ failure.^2^ A previous study suggested a scoring system (COVID-GRAM) predicting progress to critical illness, including admission to the ICU, requirement of invasive ventilation, and death in hospitalized COVID-19 patients.^3^ Including inflammatory mediators might improve the prognostic models because of their central role in the pathogenesis of severe COVID-19. Exuberant inflammation manifesting as elevated levels of cytokines, commonly referred as a “cytokine storm” has been suggested to lead to critical conditions, such as ARDS, multiorgan failure, and eventually death.

The plasma level of high mobility group box 1 protein (HMGB1), one of the damage-associated molecular pattern molecules (DAMPs), has been found to correlate with excessive cytokine storm and severity of tissue damage in patients with severe pneumonia or ARDS.^4^ HMGB1 initiates inflammation in COVID-19 patients via at least two separate pathways. The first pathway involves disulfide-HMGB1, triggering toll-like receptor-4, which causes the release of pro-inflammatory cytokines such as interleukin-6 (IL-6). The second pathway involves exogenous HMGB1, which induces the expression of the SARS-CoV-2 entry receptor (angiotensin-converting-enzyme-2 receptor) in alveolar epithelial cells in an advanced glycosylation end-product specific receptor dependent manner.^5^ In several murine models of pneumonia, targeting HMGB1 with monoclonal antibodies not only attenuates inflammatory lung injury but also decreases bacterial or viral burden in the lungs of mice.^6,7^ IL-6 is one of the main mediators of inflammatory and immune response initiated by infection or tissue injury, and increased plasma levels of IL-6 have been reported in more than half of COVID-19 patients.^8^ One recent randomized clinical trial showed that an anti-IL-6 receptor antibody, tocilizumab, can prevent the need for invasive ventilation and death in COVID-19 patients.^9^

Early identification of inflammatory mediators predicting fatal or inferior outcomes may prompt early treatment interventions, potentially yielding better outcomes in patients with severe COVID-19.^2^ In this study, we aimed to define the prognostic roles of HMGB1 and IL-6 in critically ill patients with COVID-19.

## METHODS

### Design.

The study protocol was reviewed and approved by the institutional review boards of the local ethics committees of Chonburi Hospital (129/63/S/h3) and Buddhasothorn Hospital (BSH-IRB 043/2563). Written informed consent was obtained from all patients or their first-line relative if the patient was too unwell to provide it under an emerging infectious disease provision.

Patients were treated following the Thai national COVID-19 treatment guideline from the Department of Disease Control.^10^ As a healthy control group, we analyzed stored healthy blood donors collected at pre–COVID-19 era with normal blood counts, normal values of liver enzymes, and a negative serology for viral hepatitis and HIV.

### Patients.

Sixty-seven critically ill COVID-19 patients were assessed for eligibility at admission to one of the participating ICUs, the Chonburi Hospital (Chonburi Province, Thailand) or the Buddhasothorn Hospital (Chachoengsao Province, Thailand), from January 1, 2020 through January 31, 2021. COVID-19 was confirmed by means of a positive SARS-CoV-2 real-time polymerase chain reaction assay of nasopharyngeal swabs, oropharyngeal swabs, or sputum samples. Patients were excluded if aged under 18 years or pregnant, or if admission was only for palliation. The remaining 60 COVID-19 patients were enrolled and included in the final analysis. Twelve patients (20%) died in the ICU.

### Blood sampling and measurements.

Blood samples were prospectively collected at the time of ICU admission, before start of any antiviral treatment (including favipiravir, remdesivir, or lopinavir/ritonavir) or immunomodulators (which could be corticosteroids or IL-6 inhibitors). After centrifugation, plasma was stored at −80°C.

Plasma HMGB1 and IL-6 levels were determined using a commercial enzyme-linked immunosorbent assay (ELISA) kit according to the manufacturer’s instructions (HMGB1 ELISA, cat. no. E-EL-H1554, Elabscience, Houston, TX; IL-6 ELISA, catalog number E-EL-H0102, Elabscience).

Laboratory data assessed daily in ICU included white blood cell count, hemoglobin, platelet count, absolute neutrophil and lymphocyte count, arterial blood gas analysis, total bilirubin levels, serum creatinine, D-dimer, C-reactive protein (CRP), lactate dehydrogenase (LDH), and lactate level .

### Definitions.

Sepsis was defined according to the Third International Consensus Definitions for Sepsis and Septic Shock (Sepsis-3)^11^ as an infection plus organ dysfunction identified by an increase in the Sequential Organ Failure Assessment (SOFA) score of 2 points or more. Septic shock was clinically identified by sepsis patients requiring vasopressor to maintain a mean arterial pressure of 65 mm Hg or greater together with serum lactate level ≥ 2 mmol/L in the absence of hypovolemia.

ARDS was defined using the Berlin Definition^12^ in intubated COVID-19 patients or the Kigali Modification of the Berlin Definition^13^ in nonintubated COVID-19 patients.

Metabolic acidosis was defined as a serum bicarbonate < 18 mmol/L. Acute kidney injury (AKI) was diagnosed according to the Kidney Disease Improving Global Outcomes clinical practice guidelines.^14^ Hospital-acquired pneumonia (HAP) was defined as pneumonia that occurred 48 hours or more after admission and was not apparent at the time of admission. Ventilator-associated pneumonia was defined as pneumonia that presented more than 48 hours after endotracheal intubation.

### Analysis plan.

Values were expressed in numbers and proportions or means ± SD where appropriate. Continuous variables were compared using Mann-Whitney *U* test for nonnormal data distributions, and by independent *t* test for normal data distributions. Dichotomous variables were compared using Pearson’s χ^2^ test or Fisher’s exact test.

Correlations between HMGB and IL-6 concentrations at ICU admission with peak laboratory data were estimated by Spearman’s correlation. The ICU fatality prediction of HMGB levels, IL-6 levels, neutrophil/lymphocyte ratio, D-dimer level, SOFA scores, and COVID-GRAM risk scores at ICU admission were further evaluated by receiver operating characteristic curve (ROC) analysis, and areas under the curve (AUCs) were calculated. Youden Index was calculated on the basis of the ROC to help set the appropriate cutoff value as the best obtainable balance of sensitivity and specificity. Comparison of survival curves used Kaplan-Meier survival analysis, and differences were compared using the log-rank test. Multivariate analysis was performed to adjust for disparate baseline characteristics contributing to mortality.

Data were analyzed using IBM SPSS Statistics for Windows version 18.0 (IBM Corp., Armonk, NY) and GraphPad Prism version 7.0 (GraphPad Software Inc., San Diego, CA).

## RESULTS

### Patients.

Demographic and clinical characteristics are described in Table 1. ICU mortality was 20%. Care for patients was not different for survivors versus nonsurvivors with regard to antiviral treatment; nonsurvivors received an IL-6 inhibitor more often.

**Table 1:** Demographic and Clinical Characteristics of the ICU COVID-19 cohort

Characteristics	Overall cohort (*N = 60*)	ICU nonsurvivors (*n = 12*)	ICU survivors (*n = 48*)	*P* value
Variables				
Age, years	46.3 ± 14.1	64.5 ± 11.2	41.7 ± 10.7	< 0.001*
Sex, male/female	31/29	11/1	20/28	0.003*
BMI	24.79 ± 5.36	25.94 ± 6.71	24.5 ± 5	0.407
Comorbidities, *n* (%)				
Chronic kidney disease	15 (25)	8 (66.7)	7 (14.6)	0.001*
Hypertension	15 (25)	7 (58.3)	8 (16.7)	0.006*
Diabetes mellitus	11 (18.3)	6 (50)	5 (10.4)	0.005*
Stroke	6 (10)	3 (25)	3 (6.3)	0.088
COPD/asthma	2 (3.3)	0 (0)	2 (4.2)	NA
COVID19 test reason, *n* (%)				
Contact with a confirmed COVID-19 case	40 (66.7%)	12 (100%)	28 (58.3%)	0.005*
Individual sought healthcare	16 (26.7%)	0 (0%)	16 (33.3%)	0.025
Active surveillance	3 (5%)	0 (0%)	3 (6.3%)	NA
Come back from other country	1 (1.7%)	0 (0%)	1 (2.1%)	NA
COVID-19 treatment in ICU, *n* (%)				
Favipiravir	55 (91.7%)	10 (83.3%)	45 (93.8%)	0.259
Remdesivir	5 (8.3%)	3 (25%)	2 (4.2%)	0.050
Lopinavir/ritonavir	49 (81.7%)	12 (100%)	37 (77.1%)	0.099
Hydroxychloroquine	45 (75%)	10 (83.3%)	35 (72.9%)	0.712
Corticosteroid	47 (78.3%)	8 (66.7%)	39 (81.3%)	0.271
IL-6 inhibitor (tocilizumab)	9 (15%)	5 (41.7%)	4 (8.3%)	0.012
Time from onset of illness to ICU admission/ laboratory measurements, d	9 ± 2.86	8 + 4.71	9 + 2.7	0.87
Length of ICU stay, d	13.57 ± 9.18	20.25 ± 13.37	11.9 ± 7.04	0.057

BMI = body mass index; COPD = chronic obstructive pulmonary disease; IL-6 = interleukin-6; ICU = intensive care unit; NA = not applicable. Values are mean ± SD or *n* (%) unless stated otherwise. ICU nonsurvivors versus ICU survivors.

* *P* < 0.01 chi-square test for categorical data and independent *t* test for continuous data.

### Clinical parameters and laboratory results.

ICU admission clinical parameters and laboratory findings are presented in Table 2. At ICU admission, nonsurvivors experienced worse clinical parameters including SOFA scores and COVID-GRAM scores. In addition, plasma bilirubin levels, the neutrophil/lymphocyte ratio, and D-dimer levels were higher in nonsurvivors, whereas the PaO_2_/FiO_2_ ratio was higher in survivors.

**Table 2:** Clinical parameters and laboratory findings of the COVID-19 cohort

Characteristics	Overall cohort (*N = 60*)	ICU nonsurvivors (*n = 12*)	ICU survivors (*n = 48*)	*P* value
Clinical parameters				
SOFA score at ICU admission	7.6 ± 5.24	13.17 ± 3.35	6.21 ± 4.69	< 0.001*
Maximum SOFA score	11.18 ± 5.14	17.25 ± 2.22	9.67 ± 4.51	< 0.001*
COVID-GRAM score at ICU admission	129.21 ± 35.73	174.62 ± 34.2	117.85 ± 25.9	< 0.001*
ARDS severity at ICU admission				
Mild (200 < PaO2/FiO2 ≤ 300 mm Hg)	40 (66.7%)	0 (0%)	40 (83.3%)	< 0.001*
Moderate (100 < PaO2/FiO2 ≤ 200 mm Hg)	3 (5%)	0 (0%)	3 (6.3%)	NA
Severe (PaO2/FiO2 ≤ 100 mm Hg)	17 (28.3%)	12 (100%)	5 (10.4%)	**<** 0.001*
Laboratory findings at ICU admission				
Bilirubin (mg/dL)	1.62 ± 1.84	4.14 ± 2.82	0.99 ± 0.61	0.003*
PaO2/FiO2 ratio (mm Hg)	115.9 ± 92.65	92.67 ± 9.07	134.21 ± 81.63	0.025
Neutrophil/lymphocyte ratio	12.62 ± 4.87	18.17 ± 3.82	11.24 ± 4.07	< 0.001*
Creatinine (mg/dL)	2.26 ± 1.22	2.65 ± 1.29	2.04 ± 1.17	0.096
Hemoglobin (g/dL)	10.78 ± 2.3	10.95 ± 2.8	10.35 ± 2.2	0.849
LDH (U/L)	385.83 ± 140.2	457.83 ± 185.68	367.83 ± 122.27	0.134
CRP (mg/L)	129.28 ± 63.32	154.25 ± 40.42	123.04 ± 66.72	0.128
D-dimer (µg/mL)	2.47 ± 1.19	3.58 ± 0.87	2.19 ± 1.1	< 0.001*
Lactate (mmol/L)	0.97 ± 0.73	1.31 ± 0.54	0.88 ± 0.75	0.068
Respiratory supports in ICU				
HFNO	37 (61.7%)	6 (50%)	31 (64.6%)	0.508
Noninvasive ventilation	32 (53.3%)	2 (16.7%)	30 (62.5%)	0.008*
Prone position	28 (46.7%)	7 (58.3%)	21 (43.8%)	0.520
Invasive mechanical ventilation	30 (50%)	11 (91.7%)	19 (39.6%)	0.003*
Mechanical ventilation parameters in ICU				
Plateau pressure (cmH_2_O)	26 ± 6	30 ± 4	23 ± 4	< 0.001*
PEEP (cmH_2_O)	11 ± 2.96	13.58 ± 2.15	9.37 ± 2.11	< 0.001*
Driving pressure (cmH_2_O)	15.06 ± 3.97	16.83 ± 3.76	13.95 ± 3.76	0.046*
Tidal volume (mL/PBW)	8.08 ± 0.89	8.66 ± 0.29	7.71 ± 0.94	< 0.001*
Ventilator-free days	7.52 ± 4.2	1.42 ± 3.06	9.04 ± 2.84	< 0.001*
Complications in ICU				
Metabolic acidosis-(HCO_3_^–^ < 18 mmol/L)	28 (46.7%)	12 (100%)	16 (33.3%)	< 0.001*
Pulmonary embolism	5 (8.3%)	3 (25%)	2 (4.2%)	0.050
Septic shock	21 (35%)	12 (100%)	9 (18.8%)	< 0.001*
HAP/VAP	17 (28.3%)	8 (66.7%)	9 (18.8%)	0.002*
Renal replacement therapy	6 (10%)	3 (25%)	3 (6.3%)	0.088
Pulmonary edema	14 (23.3%)	6 (50%)	8 (16.7%)	0.024*
Acute kidney injury	17 (28.3%)	12 (100%)	5 (10.4%)	< 0.001*
Maximum norepinephrine (µg/kg/min)	0.1 ± 0.14	0.27 ± 0.2	0.05 ± 0.06	< 0.001*

ARDS = acute respiratory distress syndrome; CRP = C-reactive protein; ICU = intensive care unit; LDH = lactate dehydrogenase; HAP = hospital acquired pneumonia; HFNO = high-flow nasal oxygen; NA = not applicable; PEEP = positive end-expiratory pressure; PBW = predicted body weight; SOFA = Sequential (sepsis-related) Organ Failure Assessment; VAP = ventilator associated pneumonia. Values are mean ± SD or *n* (%) unless stated otherwise. ICU nonsurvivors versus ICU survivors.

* *P* < 0.01 chi-square test or Fisher’s exact test for categorical data and independent *t*-test or Mann–Whitney *U*-test for continuous data.

During stay in ICU, nonsurvivors more often had severe ARDS and needed invasive ventilation more than survivors, with higher plateau pressure, positive end-expiratory pressure, driving pressure, and tidal volume. The number of ventilator-free days was lower in nonsurvivors, and metabolic acidosis, shock, pneumonia, pulmonary edema, and AKI was more often seen in nonsurvivors.

### HMGB1 and IL-6 levels at ICU admission.

**Figure 1.**
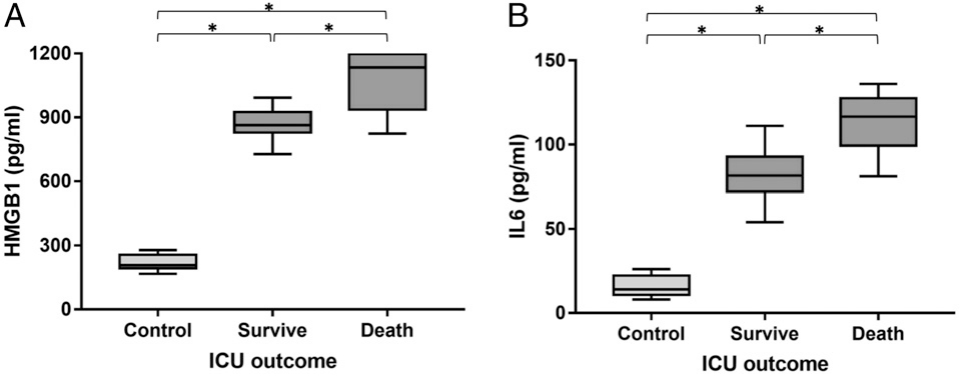
Plasma was obtained from healthy control subjects (control; *N* = 20), intensive care unit (ICU) nonsurvivors (death; *N* = 12), ICU survivors (survive; *N* = 48). (**A**, **B**) high mobility group box 1 (HMGB1) and interleukin-6 (IL-6) levels were elevated in death and survive group compared with control group, with an increase observed between death and survivor group. HMGB1 and IL-6 levels were highest in death COVID-19 patients. * *P* < 0.001 ANOVA test with Bonferroni correction.

HMGB1 and IL-6 levels were elevated in COVID-19 patients (*n* = 60) compared with healthy subjects (Figure 1 and Supplemental Table 1). HMGB1 levels (1065.2 ± 142.79 versus 871.2 ± 162.72 pg/mL, *P* < 0.001) and IL-6 levels (113.1 ± 38.43 versus 82.5 ± 39.84 pg/mL, *P* < 0.001) were higher in nonsurvivors than in survivors.

**Figure 2.**
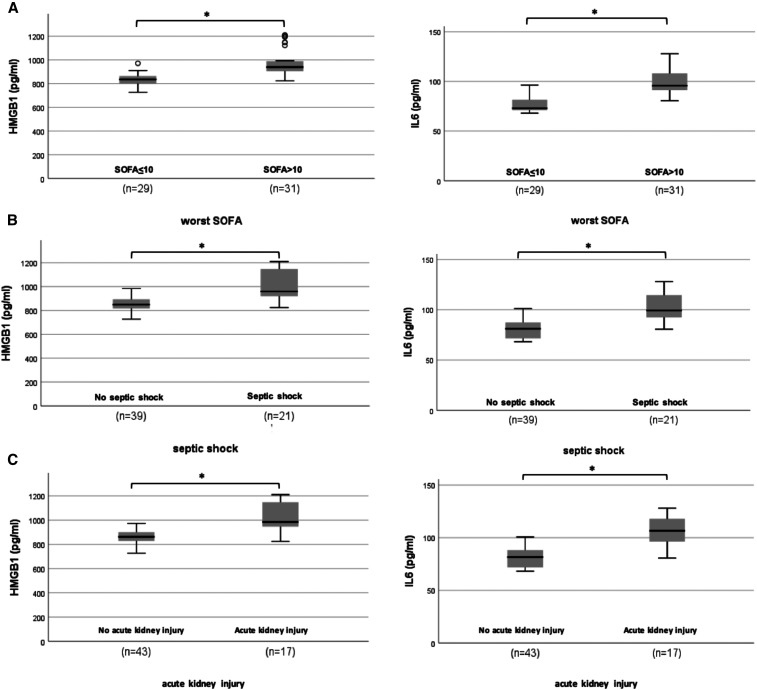

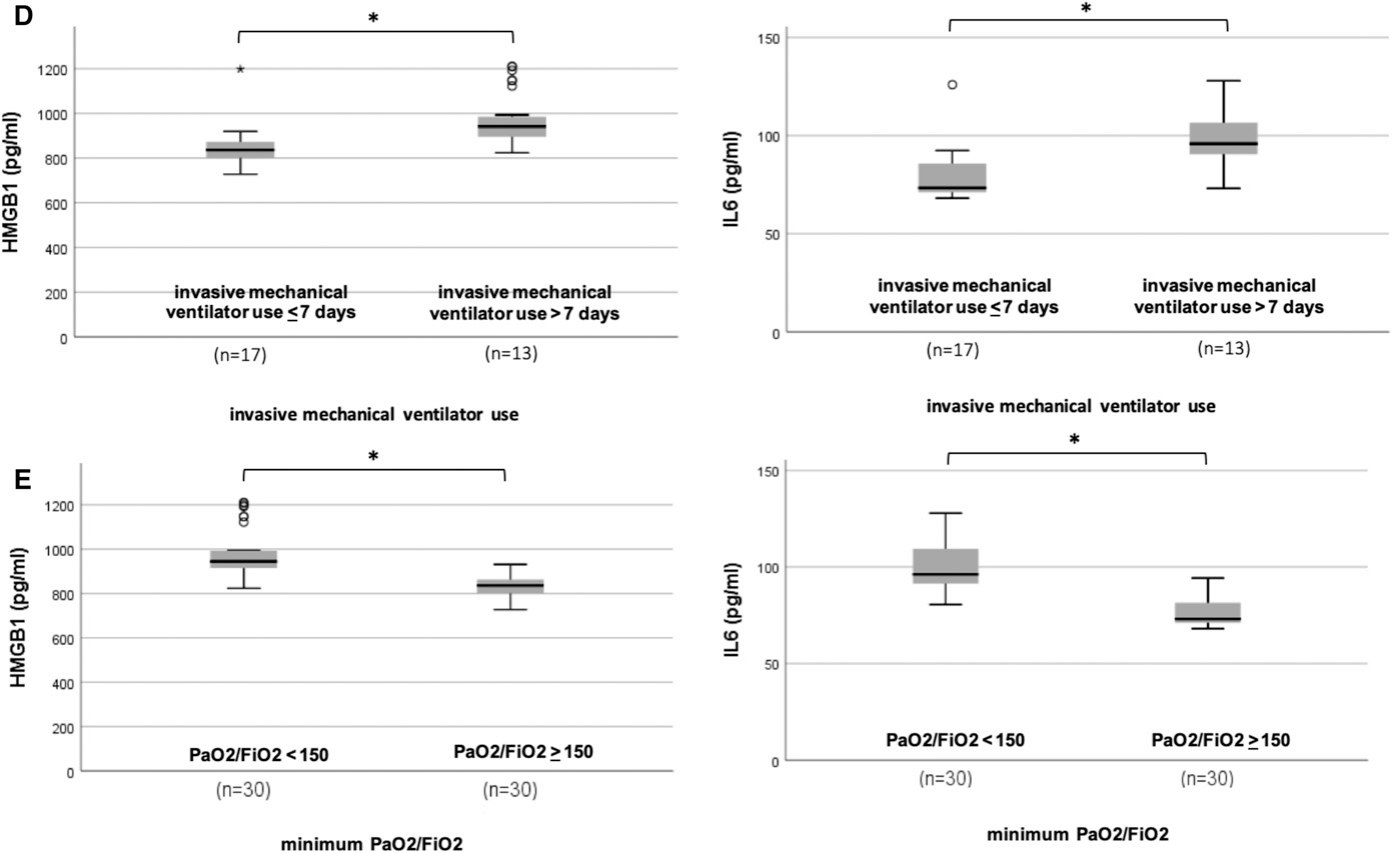
High mobility group box 1 (HMGB1) and interleukin-6 (IL-6) plasma levels in COVID-19 patients at intensive care unit (ICU) admission were significantly higher in ICU COVID-19 patients with (**A**) worse organ failure, as defined by a Sequential Organ Failure Assessment score > 10; (**B**), presence of septic shock; (**C**) presence of acute kidney injury; (**D**) a prolonged duration of mechanical ventilation, as defined by invasive mechanical ventilation use > 7 days; 2E, worse respiratory parameters, as defined by lower level of minimum PaO2/FiO2 (< 150) in ICU. * *P* < 0.001 Independent t-test.

HMGB1 and IL-6 levels correlated with all defined clinical outcomes during stay in ICU (Figure 2), and with peak neutrophil/lymphocyte ratios (r_s_ = 0.375, *P* = 0.038), peak CRP levels (r_s_=0.357, *P* = 0.049) and peak D-dimer levels (r_s_ = 0.352, *P* = 0.006) (Supplemental Table 2).

### Prognostic value of HMGB1 and IL-6 plasma levels.

**Figure 3.**
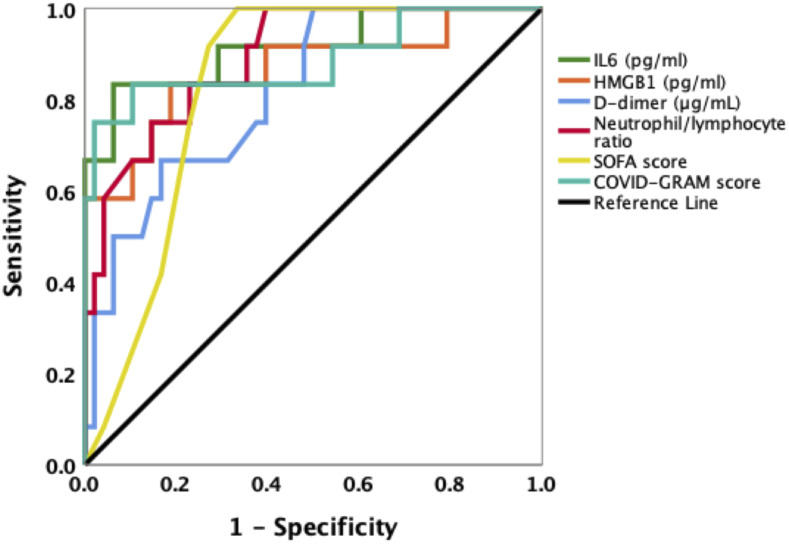
Receiver operating characteristic (ROC) curves in predicting intensive care unit (ICU) survival based on interleukin-6 (IL-6) levels, high mobility group box 1 (HMGB1) levels, D-dimer levels, or neutrophil/lymphocyte ratio or Sequential Organ Failure Assessment (SOFA) score, or COVID-GRAM risk score at ICU admission in COVID-19 patients. Areas under curves (AUCs) for IL-6 levels, HMGB1 levels, D-dimer levels, neutrophil/lymphocyte ratio, SOFA score, and COVID-GRAM risk score were 0.915, 0.865, 0.817, 0.892, 0.841, and 0.885, respectively. This figure appears in color at www.ajtmh.org.

**Figure 4.**
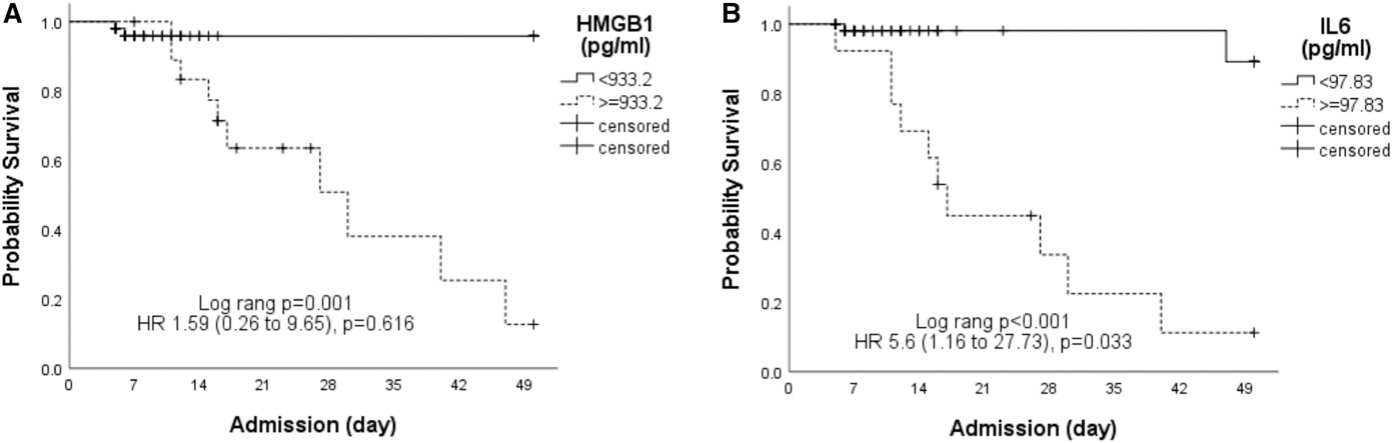
Kaplan-Meier survival curves of intensive care unit (ICU) COVID-19 patients are displayed, showing that patients with high mobility group box 1 (HMGB1) levels ≥ 933.2 pg/mL (*P* = 0.001) (**A**) or interleukin-6 (IL-6) levels ≥ 97.83 pg/mL (*P* < 0.001) (**B**) on ICU admission had a significantly higher ICU mortality compared with patients with HMGB1 levels < 933.2 pg/mL or IL-6 levels < 97.83 pg/mL.

The ROCs for HMGB1, IL-6, D-dimer, neutrophil/lymphocyte ratio, SOFA score, and COVID-GRAM risk score as a predictor for a fatal outcome in the ICU all showed high AUCs (Figure 3 and Supplemental Table 3 in Supplementary Information). The optimal cutoff for HMGB1 and IL-6 were 933.17 pg/mL and 97.83 pg/mL, respectively. Using these cutoffs, Kaplan-Meier analysis showed that patients with HMGB1 plasma levels ≥ 933.17 pg/mL or IL-6 plasma levels ≥ 97.83 pg/mL had higher ICU mortality (Figure 4). After adjustment for age, gender and comorbidities by multivariate analysis, higher levels of plasma HMGB1 and IL-6 at ICU admission were not independently associated with ICU mortality (Supplemental Table 4).

## DISCUSSION

Within this cohort of severe COVID-19 patients admitted in ICU, HMGB1, and IL-6 plasma concentrations measured at ICU admission were highest in patients with a fatal course of the disease. Plasma concentrations of these biomarkers were also higher in patients with a worse SOFA score (> 10), septic shock, or AKI and were also associated with poor respiratory outcomes, including longer times on invasive mechanical ventilator (> 7 days) and a worse PaO_2_/FiO_2_ (< 150). ROC curve analysis showed that HMGB1 and IL-6 plasma concentrations measured at ICU admission could predict subsequent death in ICU with high accuracy.

HMGB1, an important DAMP, can be actively released by innate immune cells in response to exogenous pathogens or endogenous inflammatory stimuli and can be passively released from damaged lung parenchymal cells.^15–18^ However, in contrast to classical inflammatory cytokines (e.g., tumor necrosis factor), in animal studies, HMGB1 is a late mediator of endotoxin lethality.^19^ Previous studies have shown increased HMGB1 plasma levels in critically ill patients^20,21^ but limited value as a prognosticator for death in the critically ill setting.^20,22^ This may have been caused by substantial heterogeneity in patient characteristics and inclusion of less severe patients compared with the present study. In COVID-19, it has been shown previously that HMGB1 plasma levels are elevated in severe disease,^23,24^ and are predictive of a fatal outcome, with an AUCs of 0.694.^24^ Our results are in accordance with these previous studies but show a substantial better accuracy for predicting a fatal outcome with an AUCs of 0.865. This better discrimination could be explained by the later time point of HMGB1 assessment at the moment of ICU admission (median 9 days from disease onset), rather than the moment of hospital admission in the previous study. In line with the experimental sepsis studies, HMGB1 would thus be a late mediator of a potentially fatal dysregulated host response in CVID-19. HMGB1 triggers toll-like receptor-4, generating the release of pro-inflammatory cytokine including IL-6, which is one of pathophysiological hallmarks of the cytokine storm in critically ill COVID-19 patients.^25–27^

IL-6 is well established as a biomarker for disease severity in COVID-19^28–31^ and as a predictor for adverse clinical outcomes including mortality,^32–36^ as well as for respiratory failure and the need for mechanical ventilation.^37^ IL-6 has been used as a prognostic biomarker in COVID-19-associated hyperinflammatory syndrome.^38^ In agreement with these studies, our results showed that IL-6 plasma levels at ICU admission were associated with disease severity in COVID-19 and were predictive of worse respiratory outcomes and mortality in ICU. The prognostic accuracy for a fatal outcome was higher compared with HMGB1 or D-dimer plasma concentrations, the neutrophil/lymphocyte ratio or SOFA or COVID-GRAM risk scores at admission to the ICU.

There was a significant correlation between both HMGB1 and IL-6 plasma levels at ICU admission and other prognostic laboratory biomarkers. Higher IL-6 plasma levels were associated with higher peak D-dimer and peak CRP in ICU, which can be explained by IL‐6 causing an increase acute phase reactants, such as C‐reactive protein, fibrinogen, and hepcidin.^39^ In addition, the ratio of neutrophils to lymphocytes was positively correlated with IL-6 plasma levels but not with HMGB1, suggesting IL-6 plays a more important role in the substantial reduction of the peripheral lymphocytes associated with immunoparalysis in patients with COVID-19.^36^

Our results show that both HMGB1 and IL‐6 are important biomarkers of disease severity in COVID-19 and could guide early recognition of the most severe patients at the moment of ICU admission. This could prompt increased monitoring and earlier treatment interventions, potentially yielding better disease outcomes.

The present study has limitations. First, HMGB1 and IL-6 are known to be increased in patients with acute^40,41^ or chronic kidney disease^42^ and diabetes,^43,44^ which are also conditions associated with mortality from COVID-19. This partly explains that plasma HMGB1 and IL-6 at ICU admission were not independently associated with ICU mortality after adjusting for baseline characteristics in the multivariate logistic regression model. Second, the HMGB1 and IL-6 plasma levels were prospectively measured at a single time point. A follow-up study with serial measurements and in a larger group of patients will be necessary to reevaluate the best prognostic cutoff values for HMGB1 and IL-6 and to confirm their prognostic value.

In summary the study showed that plasma concentrations of HMGB1 and IL-6 assessed at ICU admission could accurately identify COVID-19 patients with a fatal outcome of the disease, with a predictive precision similar to or better than the SOFA or the COVID-GRAM risk scores. Future studies should particularly focus on the practical clinical value of HMGB1 and IL-6, including developing a scoring system with plasma HMGB1 and IL-6 as biomarkers for early recognition of COVID-19 patients at risk for developing severe disease. In addition to IL-6, HMGB1 might also be a potential therapeutic target in severe COVID-19.

## Supplemental information, tables, and figure

Supplemental materials
