# Supplementary material for: High Mobility Group Box 1 and Interleukin 6 at Intensive Care Unit Admission as Biomarkers in Critically Ill COVID-19 Patients
Source: Am J Trop Med Hyg. 2021 May 3;105(1):73–80. doi: 10.4269/ajtmh.21-0165 (PMC8274790; doi:10.4269/ajtmh.21-0165)
Supplement: Supplementary file 1 [file tpmd210165.SD1.pdf]

SUPPLEMENTARY INFORMATION

Table S1

HMGB1 and IL-6 plasma levels in ICU non-survivors as compared with ICU survivors and control

Cytokines	ICU non-survivors (*n=12*)	ICU survivors (*n=48*)	Control (n=20)	p-value
IL6	113.1 ± 38.43	82.5 ± 39.84	16.1 ± 7.31	<0.001*[a,b,c]
HMGB1	1065.2 ± 142.79	871.2 ± 162.72	218 ± 96.26	<0.001*[a,b,c]

* ICU non-survivors vs ICU Survivors (a), ICU non-survivors vs control (b), ICU survivors vs control (c), P<0.001 (Mann–Whitney U-test with Bonferroni correction).

Table S2 Correlations of HMGB1 and IL-6 plasma concentrations at ICU admission day with peak laboratories during stay in ICU.

Laboratories during ICU admission	IL-6 (pg/ml)		HMGB1 (pg/ml)	
	r	p	r	p
Neutrophil/lymphocyte ratio	0.375	0.038*	0.304	0.097
Peak D-dimer level (µg/mL)	0.352	0.006*	0.367	0.042*
Peak LDH level (U/L)	0.324	0.075	0.317	0.082
Peak CRP level (mg/L)	0.357	0.049*	0.304	0.097

*P<0.05 (Spearman rank correlation test, only significant results are shown)

Table S3 The prognostic values

The ICU fatality prediction of IL-6 levels, HMGB1 levels, D-dimer levels, neutrophil/lymphocyte ratio, SOFA score and COVID-GRAM risk score at ICU admission were further evaluated by receiver operating characteristic curve (ROC) and their areas under curves (AUCs) were calculated. Youden index was calculated on the basis of the ROC to help set the appropriate cut-off value.

Variables at ICU Admission	ROC curve		Risk factor cutoff characterization					
	AUC	95% CI	Cut off	Sensitivity	Specificity	PPV	NPV	Accuracy
IL6	0.915	(0.811, 1.0)	≥97.83	83.3%	93.8%	76.9%	95.7%	91.7%
HMGB1	0.865	(0.729,1.0)	≥933.17	83.3%	81.3%	52.6%	95.1%	81.7%
D-dimer	0.817	(0.694,0.940)	≥2.35	91.7%	52.1%	32.4%	96.2%	60.0%
Neutrophil/	0.892	(0.801,0.984)	≥11.87	91.7%	62.5%	37.9%	96.8%	68.3%

lymphocyte ratio								
SOFA score	0.841	(0.760,0.952)	≥9.00	91.7%	68.8%	42.3%	97.1%	73.3%
COVID-GRAM score	0.885	(0.753,1.0)	≥156.60	83.3%	89.6%	66.7%	95.6%	88.3%

Table S4 Multivariate analysis of plasma IL-6 and HMGB1 levels at ICU admission and baseline characteristics in ICU non-survivor and ICU survivor patients with COVID-19.

**Variables**	**Adjusted OR**	**95% CI**	***P*-value**
Multivariate analysis			
Age (years)	1.336	0.91-1.96	0.139
Sex, male	1.000	0.00-1.00	0.875
Diabetes mellitus	3.265	0.06-191.30	0.569
Chronic kidney disease	5.999	0-4525.53	0.596
Hypertension	160.716	0.05-567768.06	0.223
IL6 (pg/ml)	1.169	0.9-1.52	0.246

**Variables**	**Adjusted OR**	**95% CI**	***P*-value**
Multivariate analysis			
Age (years)	1.417	0.9-2.28	0.131
Sex, male	1.000	0.00-1.00	0.909
Diabetes mellitus	12.289	0.02-47.25	0.952
Chronic kidney disease	0.288	0.06-37676.36	0.257
Hypertension	91.161	0.13-5987613.84	0.131
HMGB1 (pg/ml)	1.020	0.98-1.03	0.590
